# Rare multifocal emphysematous osteomyelitis as a complication of metastatic rectal cancer

**DOI:** 10.1093/jscr/rjaf890

**Published:** 2026-01-13

**Authors:** Todd P Webb, Phu Dang, Jourdan Waddell, Brianna Taylor, Mark Kiefer, Stuart Hoff, Zhamak Khorgami

**Affiliations:** Department of Surgery, University of Oklahoma-School of Community Medicine, Tulsa, OK 74135, United States; Department of Surgery, University of Oklahoma-School of Community Medicine, Tulsa, OK 74135, United States; Department of Surgery, University of Oklahoma-School of Community Medicine, Tulsa, OK 74135, United States; Department of Surgery, University of Oklahoma-School of Community Medicine, Tulsa, OK 74135, United States; Department of Surgery, University of Oklahoma-School of Community Medicine, Tulsa, OK 74135, United States; Department of Surgery, University of Oklahoma-School of Community Medicine, Tulsa, OK 74135, United States; Department of Surgery, University of Oklahoma-School of Community Medicine, Tulsa, OK 74135, United States

**Keywords:** emphysematous osteomyelitis, colorectal surgery, interventional radiology

## Abstract

Emphysematous osteomyelitis (EOM) is a rare and severe subtype of osteomyelitis where infection potentiates free air into surrounding bone. Existing literature is limited, but diabetes mellitus and malignancy are described as major risk factors. We report a patient that presented with multiple perianal abscesses and was diagnosed with an advanced rectal adenocarcinoma. He was treated with abscess drainage, antibiotics, and diverting colostomy. Several weeks later he was readmitted in septic shock and was found to have extensive areas of intraosseous gas on CT imaging involving the pelvis, thoracic and lumbar spine, left femur, and several ribs bilaterally. Biopsy culture grew *Clostridium novyi*, a pathogenic obligate anaerobe. The patient was successfully treated with broad-spectrum antibiotics. Only a handful of cases of multifocal EOM exist in current literature. More research is needed to classify the relationship between metastatic cancer and EOM and to identify modifiable risk factors of this complication.

## Introduction

Emphysematous osteomyelitis (EOM) is a rare, life-threatening variant of osteomyelitis where infection with air forming organisms produce pockets of intraosseous gas. The mortality rate is up to 32%–34% [1, 2]. EOM most often occurs in the setting of uncontrolled diabetes or malignancy [1–4]. Management of EOM parallels traditional osteomyelitis, centering on broad-spectrum antibiotics with debridement when feasible [2–4]. Common pathogens include gastrointestinal species such as *Enterobacteriaceae*, especially *Escherichia coli* and Klebsiella, or *Clostridium* species [1, 2, 5, 6]. EOM most typically involves the vertebral bodies, pelvis, and femur [1, 2, 5–7]. Other common sites are the tibia and fibula [2, 3]. We report a patient with metastatic rectal cancer who developed multifocal EOM within weeks of diagnosis. Only a handful of multifocal EOM cases exist [3, 8, 9], and to our knowledge, this is the only case where such extensive dissemination originates from an endogenous source.

## Case report

**Figure 1:**
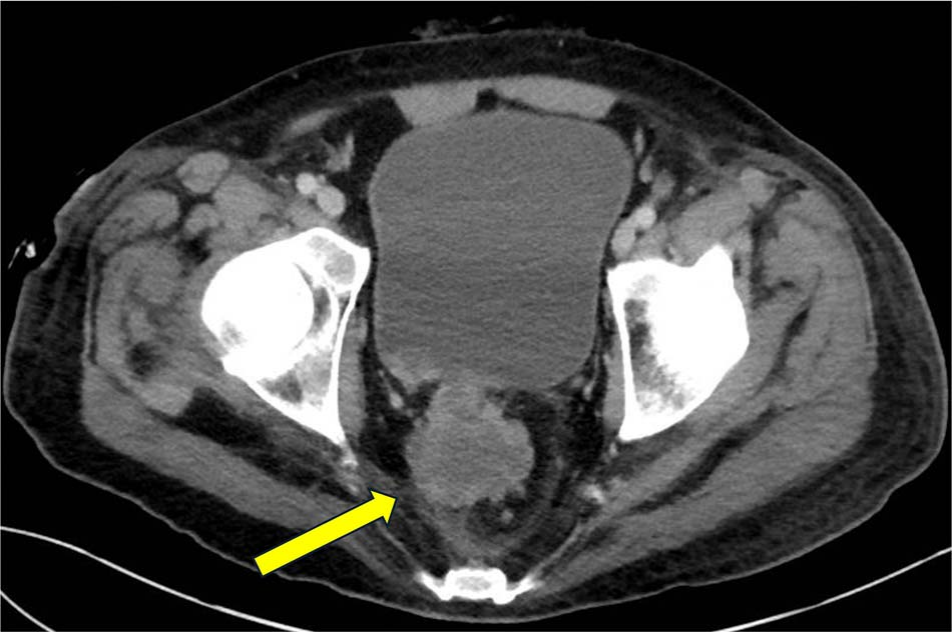
CT abdomen and pelvis—coronal plane. Primary rectal tumor measuring 4.3 × 4.5 cm.

A previously healthy 50-year-old male initially developed episodic hematochezia and “pencil-thin” stool caliber while incarcerated 1 year prior to diagnosis. Family history was notable for colon cancer in his father at age 51. The patient had not undergone recommended screening colonoscopy. He was admitted to our surgery service with multiple grossly draining perianal abscesses. Abdominopelvic imaging identified a 4.3 × 4.5 cm rectal mass with lymphadenopathy of the left internal iliac and periaortic chains (Fig. 1). Intraoperative anoscopy revealed a low rectal mass with multiple fistulizing perirectal abscesses. Broad-spectrum antibiotics were started, the abscesses were drained, and the rectal mass was biopsied, which demonstrated adenocarcinoma. The mass was determined to be unresectable, and a diverting loop colostomy was performed. Abscess cultures were polymicrobial, growing *Pseudomonas aeruginosa* and *E. coli* among other enteric anaerobes. The patient discharged on 2 weeks of narrowed oral antimicrobials and with an urgent oncology appointment but never established care. He was evaluated in our surgery clinic 1 week after discharge in stable condition with healing granulation tissue at previous fistulae tracks.

**Figure 2:**
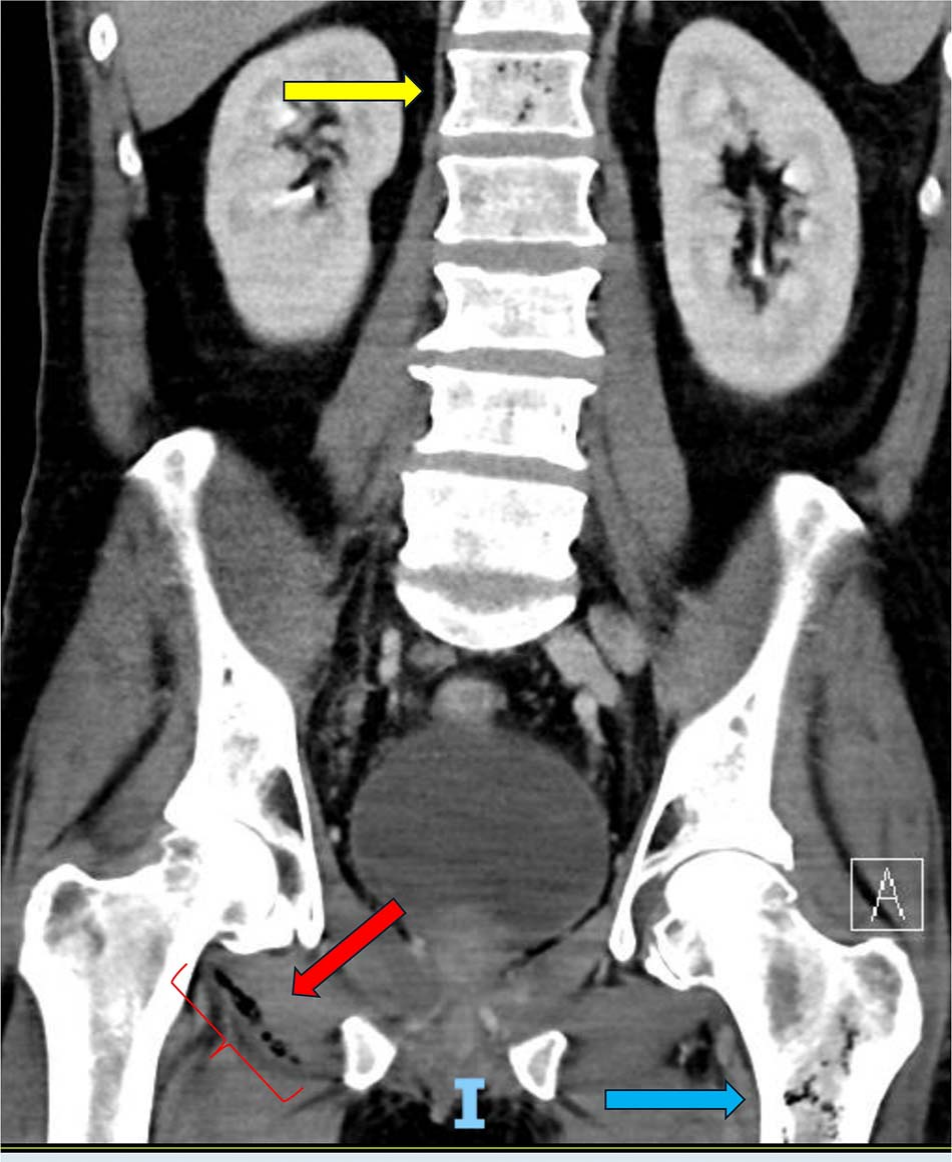
CT abdomen and pelvis—coronal plane. Intraosseous gas is visualized in the L1 vertebral body (yellow arrow, top middle) and left proximal femoral shaft to the level of the greater trochanter (blue arrow, bottom right). These sites are exemplary of the classic “pumice stone” sign, which is pathognomonic of emphysematous osteomyelitis [7]. Soft tissue gas is also seen in the right adductor longus (red arrow, bottom left). Soft tissue gas is commonly seen adjacent to involved bones in emphysematous osteomyelitis [7]. This patient has significant intraosseous gas of the right pubic ramus, although this is not visualized on this section.

**Figure 3:**
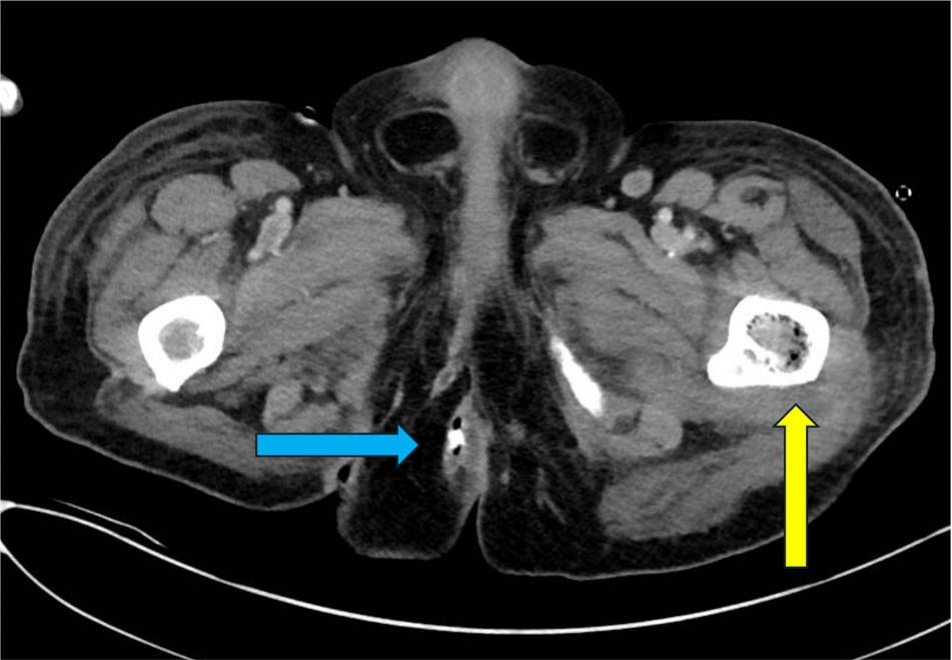
CT abdomen and pelvis—axial plane. Intraosseous gas is seen in the left femur at roughly the level of the lesser trochanter (yellow arrow, right). A previously drained perianal abscess (blue arrow, left).

**Figure 4:**
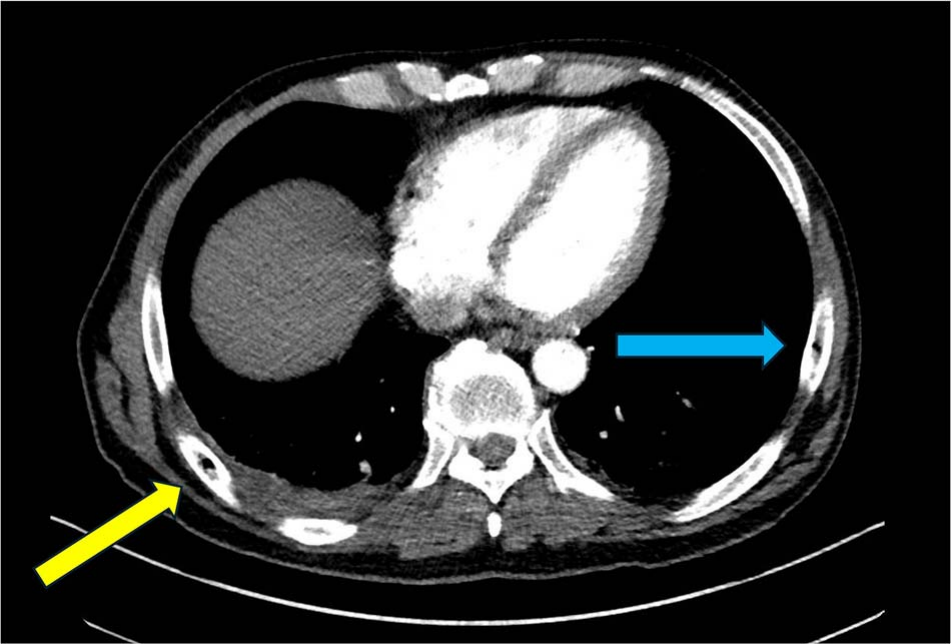
CT chest—axial plane. Intraosseous gas visualized in the right sixth rib (yellow arrow, left) and left seventh rib (blue arrow, right).

**Figure 5:**
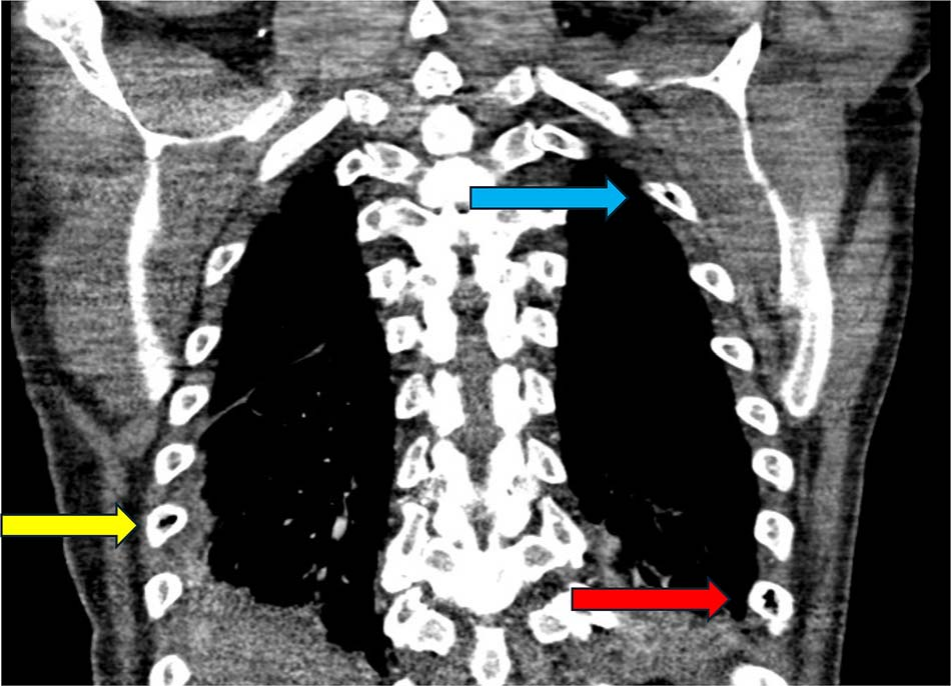
CT chest—coronal plane. Intraosseous gas is seen within the right sixth rib (yellow arrow, bottom left), left second rib (blue arrow, top right), and left eighth rib (red arrow, bottom right).

Three weeks later, the patient presented to a rural ED with fevers documented to 103.5°F (39.7°C), intractable low back pain and in severe sepsis. Hemodynamics stabilized following fluid resuscitation. He received vancomycin and meropenem prior to transfer to our facility. There was exquisite tenderness overlying the midline lumbar spine and uninfected, healing granulation tissue overlying previous perianal fistulae. His white blood cell count (WBC) was 26.21, CRP was 42.6 mg/dl (0–0.8 mg/dl) and lactate was 5.2 mmol/l (0.5–2.0 mmol/l) and glucose 99. Blood cultures were negative. CT imaging identified extensive intraosseous gas involving the right pubic ramus, sacrum, bilateral ilia, T6 and T7 vertebral bodies, L1 and L2 vertebral bodies, left proximal femur, sternum, and multiple ribs bilaterally. Soft tissue gas was additionally identified within the right obturator internus and right adductor longus (Figs 2–5). A diagnosis of EOM was made. Daptomycin, cefepime, and metronidazole were started per infectious disease.

**Figure 6:**
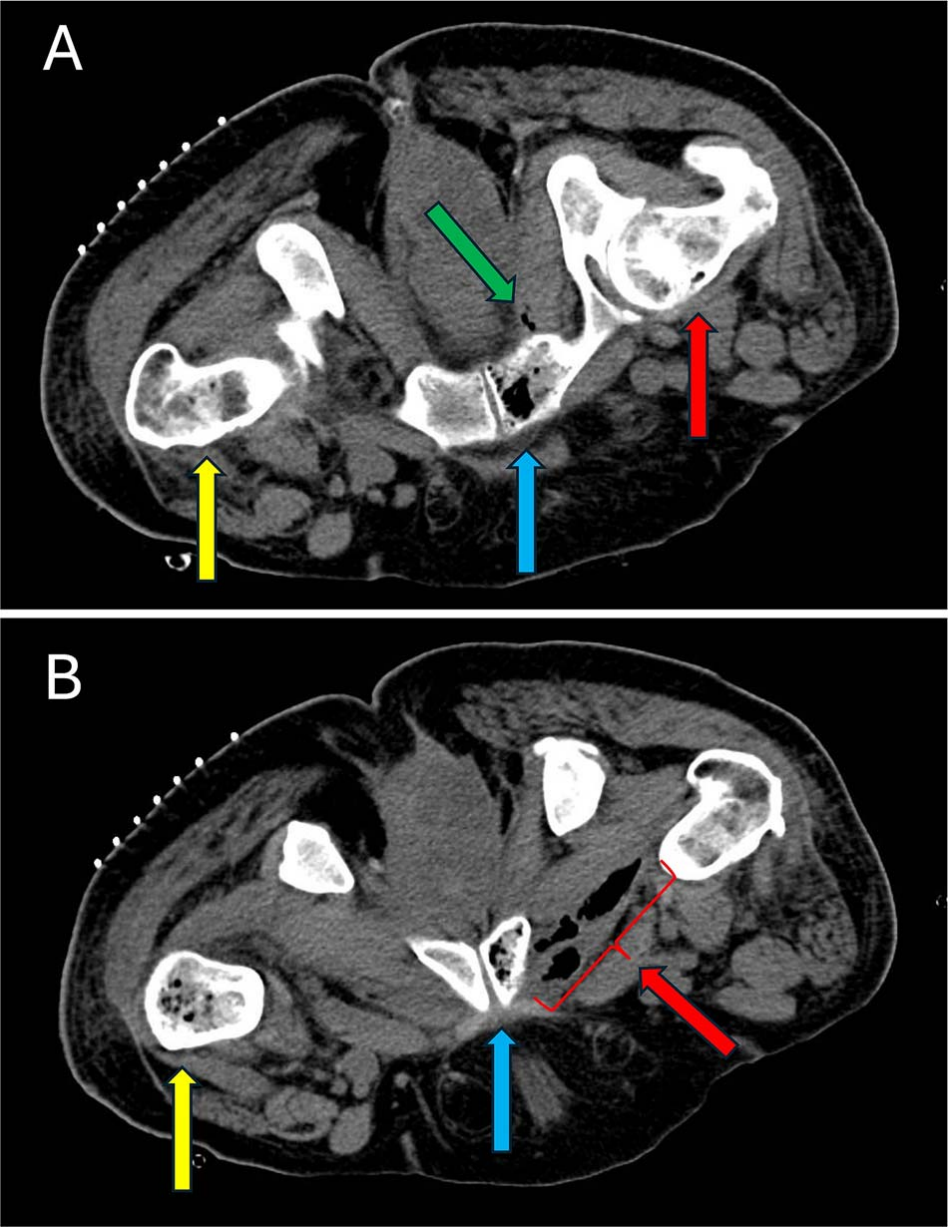
Intraprocedural fluoroscopy during left femoral bone biopsy in the axial plane with the patient in the prone position. (a) Intraosseous gas is observed in the left femoral neck (yellow arrow, left), right pubic ramus (blue arrow, bottom middle), and right femoral head (red arrow, right). Soft tissue gas is seen in the right obturator internus (green arrow, top middle) adjacent to the right pubic ramus. (b) Intraosseous gas is identified in the left proximal femoral shaft (yellow arrow, left), exhibiting a classic presentation of the “pumice stone” sign [7]. Intraosseous gas continues through the right pubic ramus (blue arrow, middle) with substantial soft tissue gas additionally identified in adjacent the right adductor longus (red arrow, right).

CT guided biopsies of the left femur and L1 vertebrae on hospital Days 1 and 4, respectively, yielded necrotic tissue without evidence of malignant cells (Fig. 6). Gram stains were positive for rare gram-negative rods, but culture of the left femoral neck grew *Clostridium novyi*, a gram-positive pathogenic obligate anaerobe, with susceptibility to metronidazole. Debridement of the lumbar spine by neurosurgery was briefly considered; however, given the extent of disease and the patient’s poor performance status this was not pursued. The patient’s WBC normalized within 2 weeks of intravenous antibiotics, and he remained afebrile, normoglycemic, and hemodynamically stable. His infectious symptoms and back pain gradually improved over several weeks.

Unexpectedly, the patient developed sudden onset left hemiplegia. During initial stroke work-up, a lesion involving the right parietal lobe was identified. A right craniotomy and tumor resection was performed with prompt resolution of neurologic deficits. Pathology confirmed metastatic rectal adenocarcinoma. Following completion of 6 weeks of daptomycin, cefepime, and metronidazole, the patient was discharged to acute rehabilitation for persisting debility but was otherwise stable.

## Discussion

This patient’s advanced rectal cancer, and subsequent bowel wall disruption, permitted hematogenous dissemination of enteric organisms. EOM classically involves the axial skeleton, with vertebral disease reported in up to 40% of cases [1]. The pelvis and proximal femur have also been frequently described [2, 3, 7]. We hypothesize that retrograde spread through the vertebral venous plexus, a valveless network connecting pelvic and vertebral veins, may explain this typical pattern of involvement in EOM [10].

In this case, it remains unclear if bony metastases preceded EOM foci. We initially suspected bone metastasis in areas of intraosseous gas, but biopsies containing necrotic tissue from two separate sites were unable to confirm or rule out malignancy. Regardless, the patient’s rapid response to antibiotics is suggestive of an infectious process. If present, metastasis likely facilitated bacterial seeding for EOM, rather than causing the finding of free air itself. Soft tissue emphysema of the right obturator internus and adductor longus, observed here, is also characteristic of EOM, as adjacent soft tissue infection is reported in 79% of cases [7]. Alternative explanations for intraosseous gas, such as degenerative arthritis, trauma, or iatrogenic introduction, were not supported by the distribution or clinical history.

*Clostridium novyi* was the only confirmed organism. Nonspecific gram-negative rods were also observed and could represent additional enteric species, like *E. coli*. The patient did receive several days of broad-spectrum antibiotics prior to biopsy, reducing culture sensitivity. However, he was critically ill on presentation, thus, empiric antibiotics took precedent over obtaining cultures. EOM carries a high mortality rate [1, 2]; thus, broad-spectrum antibiotics cannot be delayed in acutely ill patients.

To our knowledge, this case also represents the most extensive dissemination of EOM from an endogenous source reported in the literature [3, 8, 9]. Only one other case of comparable multifocal disease has been described. However, in that instance, infection was attributed to self-injection with feces in the context of significant medical and psychiatric comorbidities [8].

This report underscores that advanced rectal cancer can predispose to widespread EOM. We propose that the classic distribution of EOM along of the vertebral column, pelvis, and proximal femur is in part from spread in the vertebral venous plexus. Further study is needed to clarify the relationship between cancer and EOM, to identify modifiable risk factors that predispose to this devastating complication.
